# Visualization of Partial Exocytotic Content Release and Chemical Transport into Nanovesicles in Cells

**DOI:** 10.1021/acsnano.2c00344

**Published:** 2022-02-21

**Authors:** Tho Duc Khanh Nguyen, Lisa Mellander, Alicia Lork, Aurélien Thomen, Mai Philipsen, Michael E. Kurczy, Nhu T.N. Phan, Andrew G. Ewing

**Affiliations:** † Department of Chemistry and Molecular Biology, University of Gothenburg, Gothenburg SE-412 96, Sweden; ‡ Department of Chemistry and Chemical Engineering, Chalmers University of Technology, Gothenburg SE-412 96, Sweden; § DMPK, Research and Early Development, Cardiovascular, Renal and Metabolism (CVRM), BioPharmaceuticals R&D, AstraZeneca, Gothenburg S-431 83, Sweden

**Keywords:** nanoimaging, nanosims, partial release, exocytosis, fraction of neurotransmitter release

## Abstract

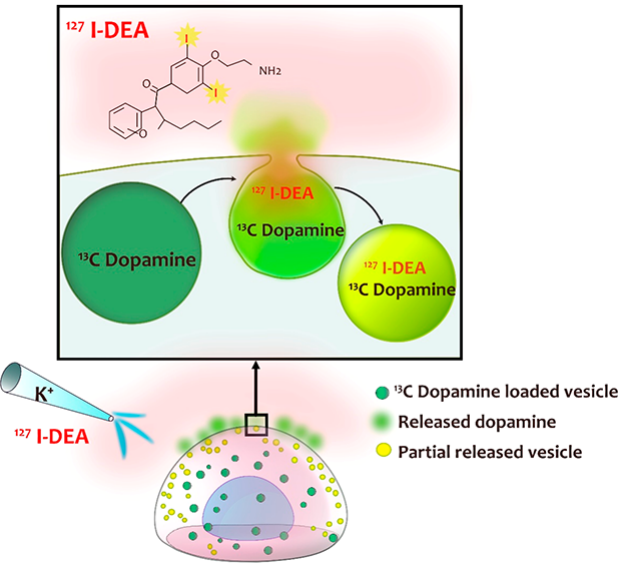

For decades, “all-or-none” and “kiss-and-run” were thought to be the only major exocytotic release modes in cell-to-cell communication, while the significance of partial release has not yet been widely recognized and accepted owing to the lack of direct evidence for exocytotic partial release. Correlative imaging with transmission electron microscopy and NanoSIMS imaging and a dual stable isotope labeling approach was used to study the cargo status of vesicles before and after exocytosis; demonstrating a measurable loss of transmitter in individual vesicles following stimulation due to partial release. Model secretory cells were incubated with ^13^C-labeled l-3,4-dihydroxyphenylalanine, resulting in the loading of ^13^C-labeled dopamine into their vesicles. A second label, di-*N*-desethylamiodarone, having the stable isotope ^127^I, was introduced during stimulation. A significant drop in the level of ^13^C-labeled dopamine and a reduction in vesicle size, with an increasing level of ^127^I^–^, was observed in vesicles of stimulated cells. Colocalization of ^13^C and ^127^I^–^ in several vesicles was observed after stimulation. Thus, chemical visualization shows transient opening of vesicles to the exterior of the cell without full release the dopamine cargo. We present a direct calculation for the fraction of neurotransmitter release from combined imaging data. The average vesicular release is 60% of the total catecholamine. An important observation is that extracellular molecules can be introduced to cells during the partial exocytotic release process. This nonendocytic transport process appears to be a general route of entry that might be exploited pharmacologically.

Exocytosis is a major neuronal communication process where the vesicles packed with signaling molecules dock, prime, fuse to the cell membrane, and release their contents. The release of the chemical messenger has traditionally been believed to be all-or-none or full release, thereby assuming an irreversible fusion and pore opening where the vesicle secretes its entire content during exocytosis.^1,2^ Later, a competing hypothesis was introduced called “kiss-and-run”^3,4^ in which exocytosis is considered to take place with an initial fusion pore that flickers to release a very small fraction of the neurotransmitter and rapidly closes.^5−7^ Over time, it has been better understood that exocytosis is a highly complicated and well-regulated process involving the role of several proteins and membrane lipids.^8−10^ For example, the SNARE-complex is known to be responsible for mediating the fusion of the vesicles with the cell membrane;^11,12^ dynamin and actin regulate the opening, expansion, constriction, and closure of the fusion pore.^13−16^ At this point, it becomes difficult for either “all-or-none” or “kiss-and-run” theory alone to explain the process as more evidence shows that the fusion pore often closes again after a significant fraction of the vesicle content is released, indicating a predominant release mode called partial release.^17−21^ The process of partial release has also been described under different names such as open and closed,^18^ subquantal release,^14^ or selective secretion.^22^

Four lines of evidence presented during the past decade support partial release. First, the total content of a secretory vesicle is greater than the amount released. The development of two analytical methods, vesicle impact electrochemical cytometry (VIEC)^23^ and intracellular vesicle impact electrochemical cytometry (IVIEC),^24,25^ by the Ewing group since 2015 allows one to obtain the total vesicle content from both isolated vesicles and vesicles in situ, respectively. The combination of VIEC/IVIEC and single-cell amperometry (SCA),^26,27^ a technique that provides quantification of neurotransmitters released from individual vesicles, has revealed that a secretory vesicle only releases a fraction of its cargo during exocytosis. The extent of partial release is speculated to depend on the intracellular calcium concentration,^28^ which can be manipulated by the strength of stimuli or pharmaceuticals. It was initially reported that the fraction of transmitter release was ∼40% for a typical exocytotic event in pheochromocytoma (PC12) cells,^29^ ∼65% in l-3,4-dihydroxyphenylalanine (L-DOPA) treated PC12 cells,^24^ ∼58% to 83% during first–fourth repetitive stimuli in PC12 cells,^30^ ∼52% in adrenal chromaffin cells when external ATP was absent, and 85% after high ATP concentration incubation,^31^ ∼80% in human carcinoid BON cells,^32^ ∼34% in pancreatic beta-cells,^33^ and ∼10% in a living *Drosophila* larval neuromuscular neuron.^19^ Second, a full opening of the fusion pore may not be necessary for quantal release. Mathematical models of the initial fusion pore size showed that the final pore opening angle is only required to be approximately 10° at maximum^34^ to release its contents, indicating that the vesicle is sealed from the extracellular environment at the end of a release event. Third, the majority of exocytotic events are followed by rapid endocytosis, a nonclathrin-dependent mechanism. A previous study reported a significant overlap at the rate between exocytosis and endocytosis;^35^ additionally, another recent study showed synaptic vesicles could participate in recycling up to a few hundred times during ∼24 h,^36^ indicating a vesicular retrieval mechanism that allows vesicles to remain structurally intact after exocytosis. Fourth, the fusion pore closing in the partial release process of vesicles was visualized by fluorescence microscopy. This has recently been supported by the works on ∼1 μm Ø vesicles in pancreatic beta-cells, and adrenal chromaffin cells using total internal reflection fluorescence microscopy (TIRF))^14,22^ and super-resolution stimulated emission depletion microscopy (STED).^37^ Partial release also appears to be a rapid and economic vesicle recycling mechanism, regulating or limiting the rate of transmitter secretion, providing machinery to adjust synaptic strength and achieve synaptic plasticity in cognition, learning, and disease.

PC12 cells, derived from a pheochromocytoma of the rat adrenal medulla, synthesize and store catecholamines in their vesicles, which later can undergo calcium-dependent exocytosis upon stimulation to release their content. Here, the loading of catecholamines into the interior of the vesicle is facilitated by vesicular monoamine transporter (VMAT) expressed on the vesicle membrane.^38^ Thus, the vesicular transmitter quantal size can be increased under treatment with the dopamine metabolic precursor, l-3,4-dihydroxyphenylalanine.^24,39^ Having these properties, the rat endocrine cell line has become a popular model to study neural differentiation and neurosecretion.^40^ Though, in PC12 cells, vesicle sizes are typically in the nanoscale (∼50–200 nm Ø), with a fusion pore of a few nanometers; a single exocytotic release event often occurs at millisecond time scale, and the number of molecules released in each event broadly varies. Therefore, it is an analytical challenge to simultaneously obtain structural, functional, and quantitative information in single cells and single vesicle analysis.

To date, the combination of electrochemical techniques with high sensitivity and high temporal resolution, such as SCA, VIEC, and IVIEC, has been extensively used as a tool to study partial release and calculate the fraction of release. However, all the evidence thus far has been indirect and the neuroscience community at large has not yet accepted the significance of partial release during exocytosis. A nanoscopic approach to visualize partial release with high spatial resolution and acquire chemical information with high sensitivity is needed. This is where a mass spectrometry imaging (MSI) technique like nanoscale secondary ion mass spectrometry (NanoSIMS), one of the very few MSI techniques that can achieve a spatial resolution down to 50 nm, becomes extremely useful. In NanoSIMS, two common primary ion sources are used, Cs^+^ and O_n_^–^, for analyzing negative ions and positive ions, respectively.^41^ The primary ions erode the sample surface, causing atomic collisions, producing highly fragmented secondary ions with limited molecular information; then the secondary ions are collected and identified based on mass-to-charge ratio. Although providing high spatial resolution, high mass resolution, and high sensitivity, at first, NanoSIMS was mainly used in geochemistry and material science. Soon after, the technique showed great potential in the analysis of biological samples,^42^ and it has proven useful for visualizing the distribution of stable isotope-labeled molecules in cells and tissue.^43−46^ Transmission electron microscopy (TEM) was initially used to achieve excellent resolution images providing vesicle morphological information and later became an essential step in sample preparation for NanoSIMS imaging. With recent developments, NanoSIMS is revolutionizing our ability to localize vesicles containing ^13^C-labeled dopamine in a single cell^47^ and yet achieve absolute quantification of neurotransmitters at a subcellular level.^48^

Seeing is believing. In this paper, we studied exocytosis in the search for direct evidence of partial release of exocytotic content and transport out of and into vesicles via the open fusion pore. To do so, we utilized correlative imaging combining TEM and NanoSIMS imaging to investigate the vesicles before and after triggering exocytosis in the cell. A dual stable isotope labeling approach was carried out by first incubating PC12 cells with ^13^C-labeled L-DOPA, resulting in the loading of ^13^C-labeled dopamine into the vesicles; later, the second label containing di-*N*-desethylamiodarone, a drug that naturally contains ^127^I (^127^I-DEA), was introduced during stimulation. The iodine allowed us to use NanoSIMS to track the fate of this molecule in the vesicles before and after release events. As a result, we enabled visualization of partial release, revealing that the vesicles that underwent partial release shrunk in size and contained a decreased level of ^13^C-labeled dopamine (release) but an increased level of ^127^I (diffusively transported into the vesicle via the transiently open pore during release). Single-cell amperometry experiments were also carried out to investigate any possible effects of di-*N*-desethyl amiodarone on the release process to confirm its validity as the second probe. Furthermore, to quantify the extent of partial release, we derived an equation that allows the direct calculation of the fraction of neurotransmitter release from the abundance measurement δ^13^C‰ and the vesicle size measurement, previously achieved only by combining data from electrochemical techniques. With this approach, we have studied the cellular process of exocytosis while showing that the partial release process allows molecular movement both out of and into nanometer vesicles during this process. Thus, this work provides a mechanism for transport of chemical species into the cell via transport into vesicles through the open fusion pore during partial release and is likely to change our understanding of chemical transport into cells.

## Results and Discussion

### Experimental Design from Single Cells to NanoSIMS Images and Quantitative Data

**Figure 1:**
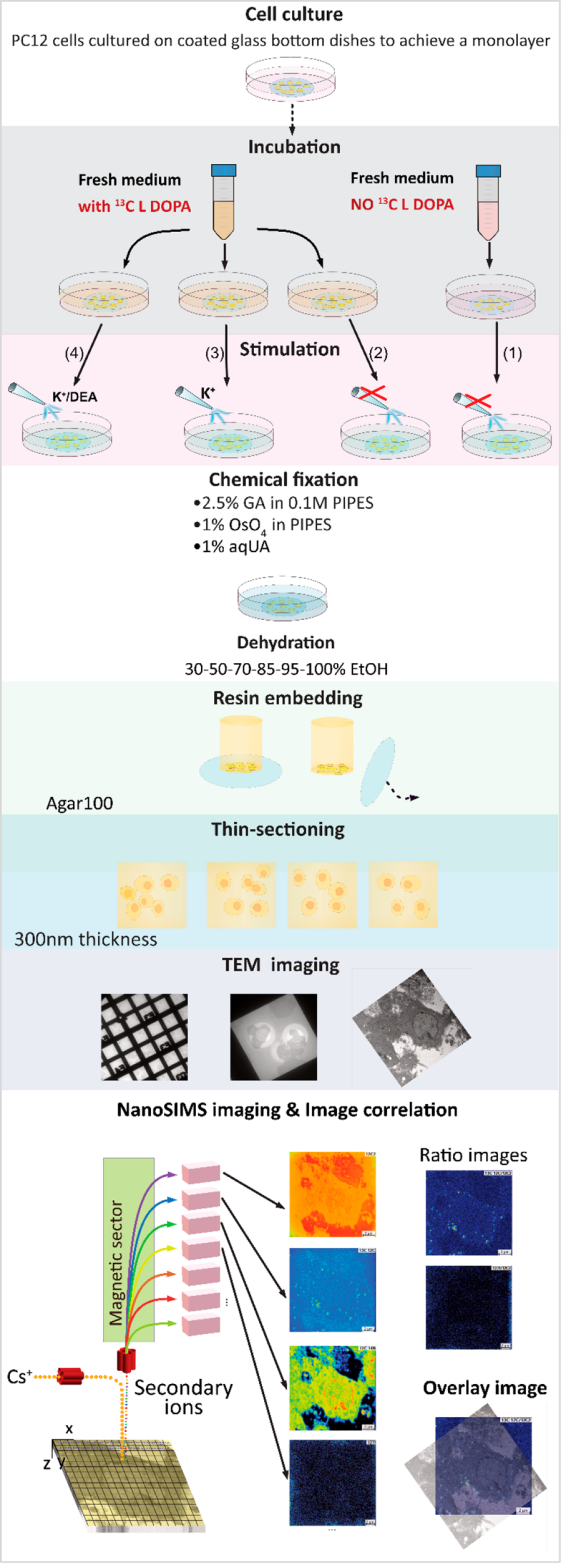
Scheme for cell preparation protocol. The workflow is described in the text.

NanoSIMS chemical imaging was carried out in PC12 cells containing isotopically labeled vesicles. Figure 1 shows the experimental design, including major sample preparation steps, and illustrates the correlative imaging technique using TEM-NanoSIMS. A dual stable isotope labeling approach was implemented by first loading ^13^C-labeled dopamine into the vesicles via 6 h incubation with ^13^C L-DOPA; later, the second label ^127^I-DEA was introduced during stimulation. The cell was stimulated twice by a 5-s delivery of 100 mM K^+^ with a 2 min interval. Four groups of samples were prepared, representing different experimental conditions required to capture the fate of the vesicles before and after undergoing exocytosis for comparison. First, the control group contained cells not incubated with ^13^C L-DOPA and not stimulated with K^+^ (1). Second, the non-stim group contained cells incubated with ^13^C L-DOPA but not stimulated with K^+^ (2). Third, the stim K^+^ group contained cells incubated with ^13^C L-DOPA and later stimulated with K^+^ solution alone (3). And last, the stim K^+^/DEA group contained cells incubated with ^13^C L-DOPA and then stimulated with K^+^ solution in the presence of ^127^I-DEA (4). After incubation and stimulation, the cells were immediately washed and fixed sequentially with glutaraldehyde (GA), osmium tetroxide (OsO_4_), and uranium acetate (UA) to preserve their morphology and capture the intravesicular dopamine and vesicles local position. Subsequently, the samples were prepared with a TEM sample preparation method (see Methods). High-resolution TEM images focusing on single cells and vesicles were acquired prior to NanoSIMS analysis, allowing the overlay and identification of specific subcellular features onto the NanoSIMS images.

### Visualizing Single Vesicles by Correlative TEM and NanoSIMS Imaging

**Figure 2:**
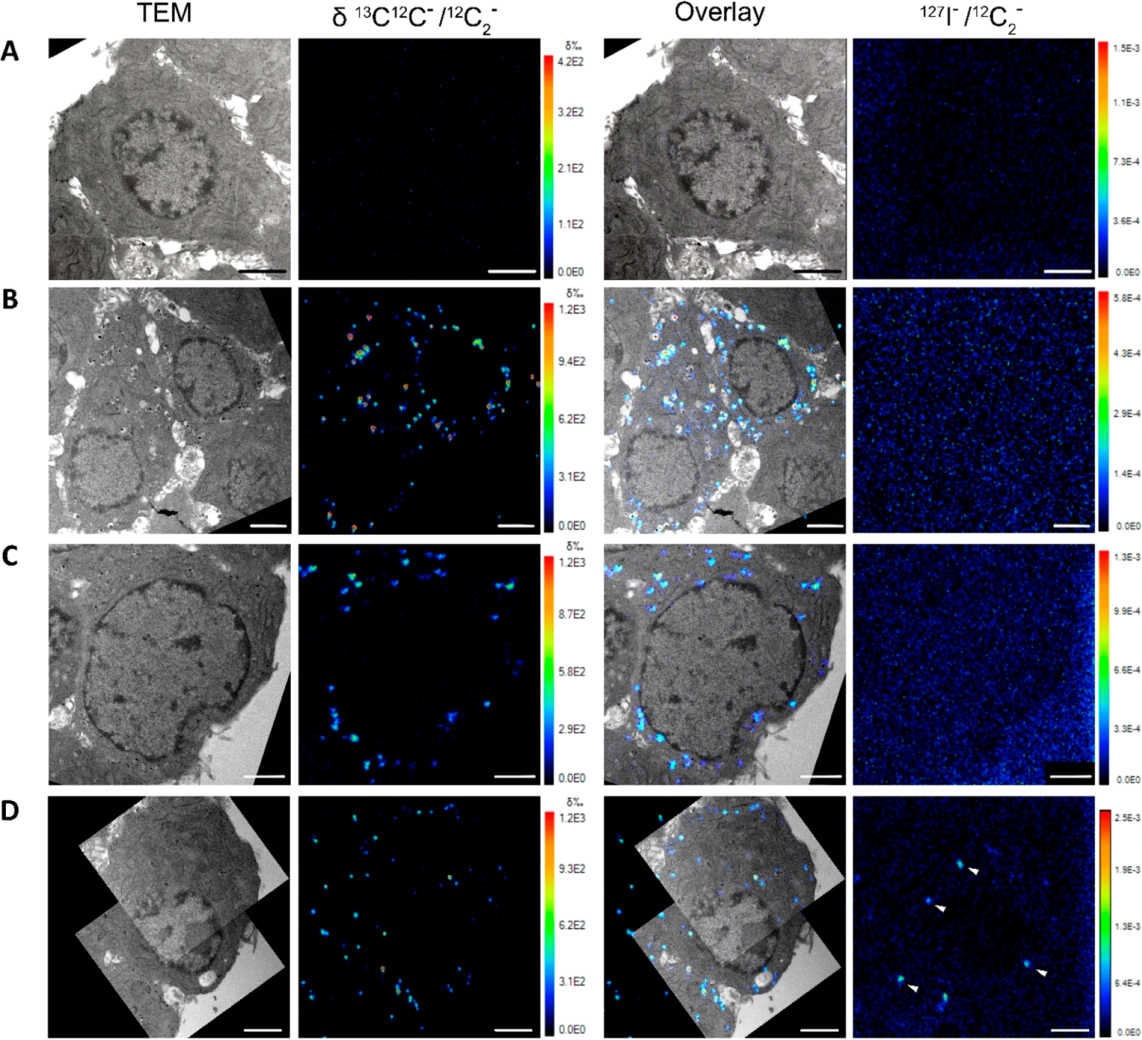
Correlative TEM and NanoSIMS ratio images of labeled vesicles in PC12 cells reveal ^13^C enrichment localized at the large dense-core vesicles and ^127^I-DEA taken up into the cells during the exocytosis process. (top to bottom panels) (A) control group (no ^13^C L-DOPA incubation, no K^+^ stimulation), (B) non-stim group (^13^C L-DOPA, no K^+^ stimulation), (C) stim K^+^ group (^13^C L-DOPA, K^+^ stimulation), and (D) stim K^+^/DEA group (^13^C L-DOPA, K^+^/DEA stimulation). (left to right) TEM images, ratio images of ^13^C^12^C^–^/^12^C_2_^–^ presented in δ‰ scale, overlay of TEM and corresponding ^13^C^12^C^–^/^12^C_2_^–^ ratio images and ratio images of ^127^I^–^/^12^C_2_^–^. ROIs representing vesicles with high ^127^I level are marked with white triangles. Scale bars: 2 μm. A series of NanoSIMS ion images of ^12^C_2_^–^, ^13^C^12^C^–^, ^12^C^14^N^–^, ^127^I^–^ used to generate the ratio images is provided in Figure S1.1. The dark line in the TEM images is the nuclear membrane with the vesicles outside this in the cytoplasm.

Figure 2 presents four panels of images for four different experimental conditions. The first panel on the left shows TEM images of PC12 cells, confirming that the cell and vesicle morphology was well preserved together with a clear visualization of the vesicles. As shown in previous studies, we also observed vesicle swelling after L-DOPA treatment.^39,47^ TEM images reveal a clear halo around the dense-core of the vesicle in the non-stim cells with ^13^C L-DOPA treatment (Figure 2B; left), while in the control cells without ^13^C L-DOPA treatment, the vesicles remain small with a smaller halo (Figure 2A; left). Interestingly, we note that in comparison with the non-stim cells, the stimulated cells from stim K^+^ and stim K^+^/DEA groups (Figure 2C–D; left) appear to have many smaller vesicles with a smaller halo, suggesting the vesicles have shrunk in size after releasing part of their content during stimulation triggering exocytosis. This is consistent with what has been proposed before: vesicular catecholamine concentration mostly remains stable when catecholamine levels are altered to maintain the osmotic equilibrium between the vesicle interior and cytoplasm, achieved by a change in the vesicular volume.^39^ We present further investigation of this point with a more dedicated vesicle size analysis later in this work.

The isotopic ratio images of ^13^C^12^C^–^/^12^C_2_^–^ presented in δ‰ scale (second panel from the left) reveal local isotopic enrichments of ^13^C-dopamine. The correlative TEM and NanoSIMS ratio images (third panel from the left) show ^13^C enrichment localized at the vesicles. As expected, untreated control PC12 cells did not show an increase in the isotopic ratio of ^13^C. In contrast, cells treated with ^13^C L-DOPA showed colocalization of ^13^C-dopamine enrichment within the vesicles (Figure 2B–D; third panel from the left). The level of ^13^C-dopamine enrichment within the cytosol of the incubated cells is close to that of the control no incubated cells. This confirms that the enrichment of ^13^C-dopamine can be used to identify the vesicles in NanoSIMS images.^47^

**Figure 3:**
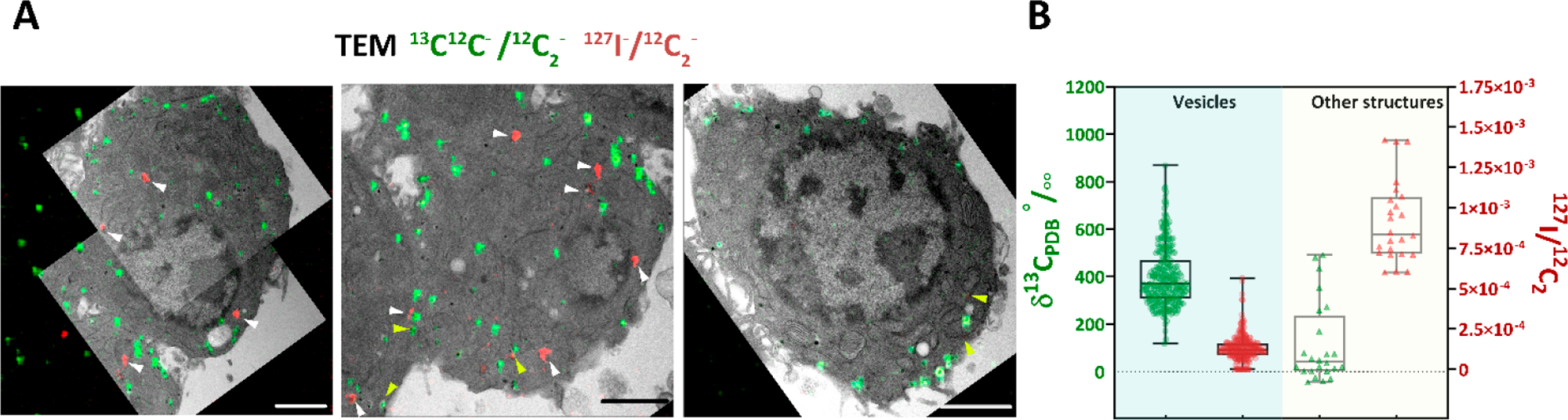
^127^I-DEA is taken up into cells during K^+^-stimulated exocytosis. (A) Correlative TEM and NanoSIMS ratio images of ^13^C^12^C^–^/^12^C_2_^–^ (in green) and ^127^I^–^/^12^C_2_^–^ (in red). Three representative images of three single cells from stim K^+^/DEA group (^13^C L-DOPA incubation, K^+^ stimulation in the presence of DEA) are shown. ROIs with high ^127^I level (hotspots/other structures) are marked with white triangles, and ROIs with high levels of ^127^I and ^13^C are marked with yellow triangles. Scale bars: 2 μm. A series of TEM and NanoSIMS ratio images including enrichment level in δ‰ scale of the three representative cell is given in Figure S1.2. (B) Box plot of δ^13^C_PDB_‰ (in green) and ^127^I^–^/^12^C_2_^–^ ratio (in red) included all ROIs (corresponding to the vesicles and the hotspots/other structures identified in TEM images) from the stim K^+^/DEA group. Data sets are presented with the box 25th–75th percentile, middle line 50th percentile, whisker boundary min to max.

The right panel shows ratio images of ^127^I^–^/^12^C_2_^–^ revealing the local distribution of ^127^I-DEA. The result confirms that the ^127^I-DEA signal can be localized within cellular structures by NanoSIMS imaging. Here, ^127^I-DEA was only introduced into the stimulation solution for the cells in the stim K^+^/DEA group but not any other groups. Thus, as expected, the ^127^I^–^ signal only increases and appears at a high level at some local hotspots within the cells of the stim K^+^/DEA group (Figure 2D; right) (Figure 3A) while staying at a much lower level (natural abundance) in other groups (Figure 2A–C; right). Additionally, during the development of the experimental design for this work, we also performed a set of samples where ^127^I-DEA was added in the isotonic solution surrounding the cells for the same time interval of 2 min; however, we did not apply K^+^ stimulation solution to trigger exocytosis. Analyzing these samples showed no significant increase of ^127^I^–^ signal within the cells (data not shown), indicating that within this short period, ^127^I-DEA does not simply transport into the cells without the initiation of stimulated exocytosis. Furthermore, although ^127^I-DEA, whose parent molecule is amiodarone, can interact and cross cellular membranes, the uptake kinetics of these molecules into cells via incubation is on a time scale of hours to days,^49^ which is slow compared to the exposure time used in this work. This strengthens the validity of ^127^I-DEA as the second label to identify vesicles that have undergone partial release upon stimulation.

Further investigating specific cellular structures in the stim K^+^/DEA group, we identified the local hotspots with a high level of ^127^I^–^, but not enriched with ^13^C; these are marked with white triangles (Figure 3A) and labeled as other structures in Figure 3B. These local hotspots appeared in TEM images to correlate with some vesicle-like structures but are larger in size and do not have a dense-core-like compartment. Therefore, we speculate that these other structures might be endosomes that have captured and transported ^127^I-DEA inside the cells during the stimulation via exocytosis followed by rapid endocytosis.

More importantly, we identified several ROIs containing a significant level of ^127^I^–^ and still highly enriched with ^13^C; marked with yellow triangles (Figure 3A) and correlated these to the vesicles in TEM images. The presence of ^127^I^–^ and ^13^C-labeled dopamine colocalized in the same vesicles strongly supports the idea that the vesicles have opened to the cell exterior secreting a fraction of their original dopamine cargo and closed again. It should be noted that the ^127^I^–^ level was also elevated in many vesicles within cells of the stim K^+^/DEA group at a moderate level; thus, further quantification analysis of the stable-isotope was carried out in the next part of the work.

### Quantification of ^13^C-Dopamine and ^127^I-DEA in the Vesicles Across Stimulated and Non-stimulated Cells

In addition to visual inspection, we quantified the ^13^C-dopamine enrichment (δ^13^C_PDB_‰) and ^127^I-DEA levels per vesicle from all four groups to obtain better insight into how the labeled vesicular content changes during exocytosis upon stimulation.

**Figure 4:**
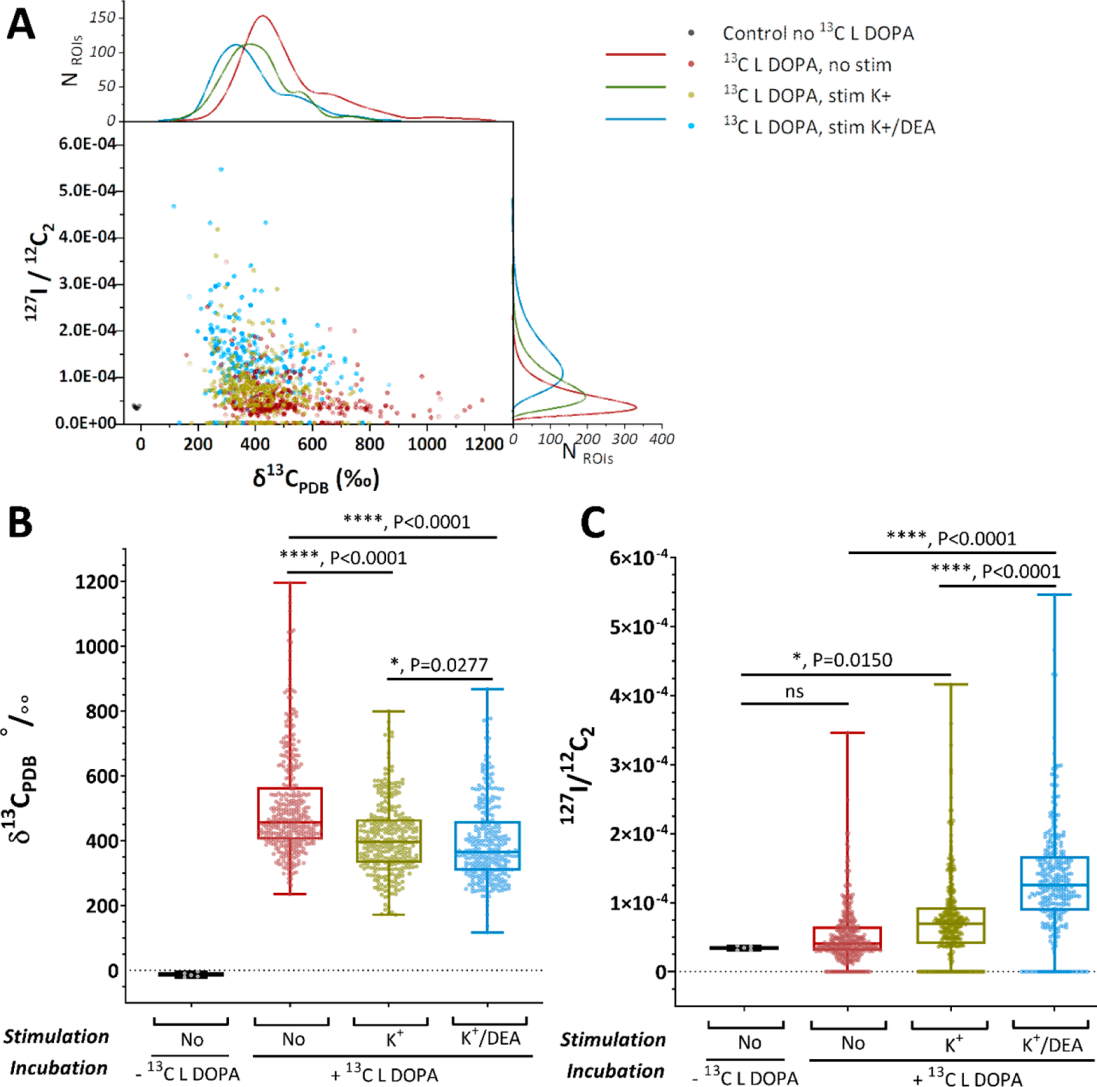
Quantitation of ^13^C-dopamine and ^127^I-DEA in vesicles. The level of ^13^C enrichment and ^127^I^–^ change upon stimulation across different groups. (A) Scatter plot with histograms showing the distributions of ^13^C enrichment level (δ^13^C _PDB_‰) and ^127^I^–^ level for all ROIs (vesicles). (B) Box plot of δ^13^C _PDB_‰. (C) Box plot of ^127^I^–^/^12^C_2_^–^ ratio across the four groups of samples. Data sets are presented with each box 25th–75th percentile, middle line 50th percentile, whisker boundary min to max, show all points. Data sets are compared with a nonparametric, two-tailed Mann–Whitney unpaired test, * *P* < 0.05, ** *P* < 0.01, *** *P* < 0.001, **** *P* < 0.0001. In the control group, four big ROIs covering different intracellular structures are analyzed, representing the background level of ^13^C. The numbers of ROIs (vesicles) analyzed are 378, 304, and 286 for the non-stim group, stim K^+^ group, and stim K^+^/DEA group, respectively.

In Figure 4A, we show a scatter plot with histograms revealing the distributions of all ROIs (vesicles) based on their ^13^C enrichment level (δ^13^C_PDB_‰) and ^127^I-DEA level (^127^I^–^/^12^C_2_^–^ ratio). In Figure 4B,C, we show the levels of ^13^C enrichment (δ^13^C_PDB_‰) and ^127^I-DEA (^127^I^–^/^12^C_2_^–^ ratio), respectively, in box plots with statistical analyses. The ^13^C-dopamine control cells (in black) showed no enrichment, and the highest level was measured in cells of the non-stim group (in red, ^13^C L-DOPA, no K^+^ stimulation). Cells in the two stimulated groups (in green, ^13^C L-DOPA, K^+^ stimulation; in blue, ^13^C L-DOPA, K^+^/DEA) showed a significant drop in the level of ^13^C-dopamine enrichment compared to the non-stim cells, while the average number of vesicles per cell in the non-stim and stim groups only changed slightly. The observation that ^13^C-dopamine remained after stimulation in stimulated cells is the first indication of partial release. This also indicates that the ^13^C level decreases due to a fraction of the total ^13^C-labeled dopamine in the vesicles being released upon stimulation. The observation that ^13^C-dopamine remains is the first indication of partial release and the small but statistically significant decrease in ^13^C-dopamine shows that the content of these vesicles has been changed following the stimulation. These data are consistent with the concept of partial release^18,20^ that has been reported in previous studies on different cells models using electrochemical techniques.^24,29−33^ Additionally, we also performed an extra set of experiments applying freeze-drying sample preparation with NanoSIMS (data shown in S4). Similarly, the results also revealed a drop in ^13^C-dopamine enrichment in the vesicles of stimulated cells compared to non-stimulated cells.

The ^127^I-DEA level in control cells (in black) and cells of the non-stim group (in red) showed no enrichment (Figure 4C). However, the iodine signal was slightly elevated in the stim K^+^ group over that in the non-stim group. We suspect this could be due to a low amount of iodine impurity present in the K^+^ stimulation solution as the KCl stimulant is only 95% pure. The highest ^127^I-DEA level was measured in cells of the stim K^+^/DEA group (in blue), and this difference is statistically significant in comparison to that of the non-stim (in red) and stim K^+^ (in green) groups. These data indicate that ^127^I-DEA introduced in the stimulation solution (for stim K^+^/DEA group) enters the vesicles during the period of time that the fusion pore opens before closing again.

Though the mechanism of how ^127^I-DEA enters the vesicles is still speculative, it seems that diffusion along the concentration gradient from relatively high outside the cell to low inside the vesicle would be sufficient. Previous studies have also shown that amiodarone, the parent molecule of ^127^I-DEA, has strong interaction with both the hydrophobic hydrocarbon chains and the polar headgroup of phospholipids.^50,51^ Thus, it is possible that after ^127^I-DEA diffuses into the vesicle through the open pore it is then anchored into the inner leaflet of the vesicles lipid bilayer. Although the focus of this work is to examine the process of partial release with imaging, these data also provide strong evidence for molecular entry into the cell via the open fusion pore during partial exocytosis.

### ^13^C-Dopamine Levels and Vesicle Size in Relation to Their Spatial Segregation

For vesicles to participate in the process of exocytosis, after being produced inside the cell, they first must be transported to be in close proximity with the target zone on the plasma membrane, then dock, prime, and fuse with membranes via specific protein–protein interactions to release their contents.^11,52^ Thus, vesicles at different stages reside at different positions in the cytoplasm during their lifetime, including those that are further away from the membrane (the reserve pool), those that are docked closely to the membrane, ready to undergo exocytosis (the releasable pool), and those that are not primed and located away from the membrane after undergoing exocytosis.^53^ Considering the observation that the ^13^C-dopamine enrichment level (Figure 4) and vesicle size (Figure 2) dropped in cells following stimulated release compared to the non-stim group, we investigated in detail the effects of vesicle position on content and size. We examined ^13^C-dopamine enrichment and vesicle size as a function of spatial segregation, including vesicles positioned close to the cell plasma membrane (outer vesicles) and those located further away from the membrane (inner vesicles). Thus, the inner vesicles are considered to represent the reserve pool while the outer vesicles include the releasable pool and the unprimed vesicles residing away from the membrane after exocytosis.

**Figure 5:**
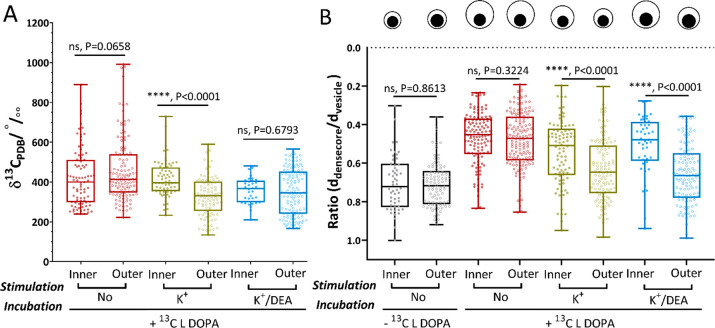
Quantification of ^13^C-dopamine and size analysis of vesicles in relation to their position. (A) Box plots of δ^13^C_PDB_‰ of inner and outer vesicles from ^13^C L-DOPA incubated cells belong to the non-stim group (in red), stim K^+^ group (in green), and stim K^+^/DEA group (in blue). (B) Box plots of the diameter ratio *d*_dense-core_/*d*_vesicle_ across the control group (in black) and the three ^13^C L-DOPA treated groups. The top panel pictorially shows a series of schematic representations of large dense-core vesicles generated based on the size measurement and *d*_dense-core_/*d*_vesicle_ of the corresponding groups (Table S2). Data sets are presented with each box 25th–75th percentile, middle line 50th percentile, whisker boundary min to max, show all points. Data sets are compared with a nonparametric, two-tailed Mann–Whitney unpaired test, * *P* < 0.05, ** *P* < 0.01, *** *P* < 0.001, **** *P* < 0.0001. Outer vesicles include those positioned within two-vesicle diameters to the plasma membrane. The numbers of vesicles measured away from the membrane, *N*_inner-vesicles_, for the four groups left to right are 54, 124, 88, and 45; for *N*_outer-vesicles_ they are 69, 144, 112, and 120.

Figure 5A shows the ^13^C-dopamine enrichment level (δ^13^C_PDB_‰) for the inner and outer vesicles across the non-stimulated and stimulated cells (with K^+^ and K^+^/DEA). Figure 5B presents the results of vesicle size analysis for the inner and outer vesicles across all four groups of cells as a ratio of dense core to vesicle diameters (*d*_dense-core_/*d*_vesicle_). As previously reported, the size of the vesicle changes after L-DOPA or reserpine treatment owing to swelling or shrinking of the halo, while the dense-core mostly remains stable.^39,47^ Thus, a smaller diameter ratio of *d*_dense-core_/*d*_vesicle_ represents a larger vesicle. Our data showed no significant difference in ^13^C-dopamine enrichment and vesicle size between inner and outer vesicles in non-stimulated cells belonging to both nonloaded and ^13^C L-DOPA loaded PC12 cells (in black and red, respectively) (Figure 5A,B). This indicate that under normal conditions without stimulation, the vesicles from either the reserve pool or the releasable pool are homogeneous and store similar amounts of dopamine. This observation is in agreement with recent research by Gu et al.^53^ and opposed to the theory of two dense-core vesicle subpools,^54^ which is not generally accepted in neuroendocrine cells. Interestingly, we found a significant decrease in ^13^C-dopamine enrichment between inner and outer vesicles in K^+^ stimulated cells (in green) (Figure 5A), meaning a fraction of the total ^13^C-labeled dopamine in the outer vesicles (in the releasable pool) had been secreted in response to a chemical stimulus. Gu et al. also proposed that vesicles undergoing partial release retrieved and located closely in the releasable pool might be transiently refilled with more transmitters, or vesicles in the reserve pool might be transported to replenish the releasable pool to some extent. However, full replenishing is very unlikely to happen within a short time period.^53^ In our case, it should be noted that cells were exposed to ^13^C-labeled L-DOPA only during the incubation time. In addition, the precursor molecules were actively converted and loaded into the vesicles via VMAT, resulting in their clearance from cell cytosol. This was confirmed by our data showing a very low level of ^13^C enrichment present in the cytoplasm of incubated cells (Figure 1, second panel from the left). As vesicles shrink and expand to maintain the inner concentration, the significantly lower level of ^13^C-labeled dopamine in stimulated cells compared to that of non-stimulated cells appears to be the result of partial release during exocytosis followed by refilling with nonlabeled dopamine. A second possibility is that as the cells were fixed immediately, the time interval might have been too short to refill/replenish dopamine in the vesicle. In both cases, the interpretation is that partial release is observed and the level of dopamine observed is proportional to the fraction released.

In terms of the vesicle size, our data showed that, in general, the vesicles of K^+^-stimulated cells (in green) shrank in comparison with non-stimulated cells (in red), and the shrinking extent is more severe for the outer vesicles compared to the inner vesicles (Figure 5B). This indicates that several of the vesicles underwent partial release. The observation of a smaller vesicle size in the inner pool as well as in the outer pool can be explained by the migration of partly emptied vesicles into the reserve pool. This has previously been described as spatial intermixing of vesicles,^55^ where vesicles in the recycling and reserve pools are thought to be spatially but not functionally intermixed.^56^

Evaluating the stim K^+^/DEA group (in blue), we observe that overall vesicles in these cells follow a similar trend to those in the stim K^+^ group, where both show a significant decrease in the level of ^13^C-dopamine enrichment and vesicle size in comparison to the non-stimulated cells. Dividing vesicles into inner and outer populations helps to reveal the difference in size, but not the ^13^C enrichment level (Figure 5A,B).

It should be noted that there is a considerable difference in the ratio of vesicle distribution for inner and outer groups compared to that of the non-stim (in red) and the stim K^+^ (in green) cells. Our data show that for the non-stim and the stim K^+^ group, this ratio is approximately 40:60 (inner: outer), whereas for the stim K^+^/DEA group, it is 20:80 (inner: outer) (Figure S3.2), indicating that more vesicles in the reserve pool (inner) are translocated closer to the membrane (outer) in the presence of ^127^I-DEA. One possible explanation is that intracellular DEA might cause an increase in local free calcium concentration around the ER through receptor-mediated channels,^57^ facilitating the transport of vesicles toward the plasma membrane. This effect might also reduce the probability that vesicles that have undergone partial release are transported further away from the plasma membrane back into the intermixing pool as observed for the stim K^+^ group. In contrast, using single-cell amperometry to investigate the effect of ^127^I-DEA on the release process, we observed that DEA reduced the number of exocytotic events during the second stimulation (Figure S5.1). As mentioned earlier, ^127^I-DEA interacts strongly with both the hydrophobic hydrocarbon chain and the polar headgroup of phospholipids.^50,51^ Thus, once it gets trapped inside vesicles and anchors deeply into the lipid bilayer,^58^ it could reduce the mobility of phospholipids needed for vesicles to rearrange their membrane lipid to form a fusion pore. We suggest that ^127^I-DEA might support the transport of vesicles from the reserve pool closer to the plasma membrane while simultaneously impairing vesicle fusion.

### Calculation of the Fraction of Release F% from TEM-NanoSIMS Data

Considering partial release as a dominant mode of exocytosis, the fraction of release F% in this context is the percentage of how much from the total number of transmitter molecules contained in a single vesicle is released during exocytosis. Knowing the fraction of release makes it possible to gain insights into the release mechanisms and understand how regulating this fraction affects the rate of transmitter secretion, synaptic strength, and plasticity. Currently, the only tool to calculate the fraction of release is combining SCA with VIEC or IVIEC data, in which the number of molecules released is achieved via SCA and the total number of molecules contained in a vesicle is achieved via VIEC/IVIEC.

Thomen et al. recently reported a method using NanoSIMS imaging to achieve absolute quantification of neurotransmitters at a subcellular level.^48^ After thoroughly investigating several aspects of the resin embedded sample and the parameter for the analysis on the NanoSIMS instrument, a penultimate equation was developed to calculate the absolute concentration of dopamine.
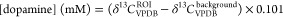
1It should be noted that the size of the vesicles changes significantly after stimulation, while the available level of ^13^C-labeled L-DOPA left in the cytosol for refilling is low, as we showed above. Therefore, this factor should be considered when calculating the number of molecules in a single vesicle in our case. Here, we employ eq 1 as a foundation and derive another formula allowing one to calculate the fraction of release directly from the abundance measurement δ^13^C‰ and the vesicle size measurement. But, first, we can calculate the number of dopamine molecules in a vesicle by using the vesicular volume and dopamine concentration as shown in eq 2, where *N*_Avogadro_ is Avogadro’s number
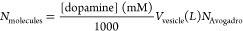
2Assuming vesicles are spherical, we now can use the vesicle diameter, *d*_vesicle_, which can be acquired from high-resolution TEM images, to replace volume, *V*_vesicle_, giving eq 3.
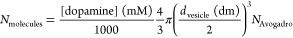
3Substituting the equation for [dopamine] (mM) in eq 1 into eq 3, we have eq 4.
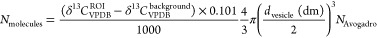
4Based on the definition given above, F% is calculated in eq 5.
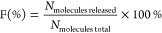
5However, we obtain the number of molecules before and after the release upon stimulation with NanoSIMS data; thus eq 5 can be transformed into eq 6.
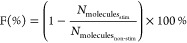
6Substituting the equation for *N*_molecules_ in eq 4 into eq 6, the fraction of release F% is obtained in eq 7.
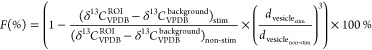
7We note here that in eq 7, it becomes the ratio of the corrected abundance δ^13^C and the ratio of the vesicles size needed for the calculation. In the work of Thomen et al., during method validation, the authors also showed that the dopamine concentration acquired is closer to the value reported in electrochemistry when the smaller aperture diaphragm (D1_5) instead of the standard one (D1_3) was used, due to the reduction in beam mixing. Here, by using the ratio of the corrected abundance δ^13^C (eq 7), as both stimulated and non-stimulated samples were analyzed with the same diaphragm, this effect on the calculated faction of release F% can be neglected.

**Figure 6:**
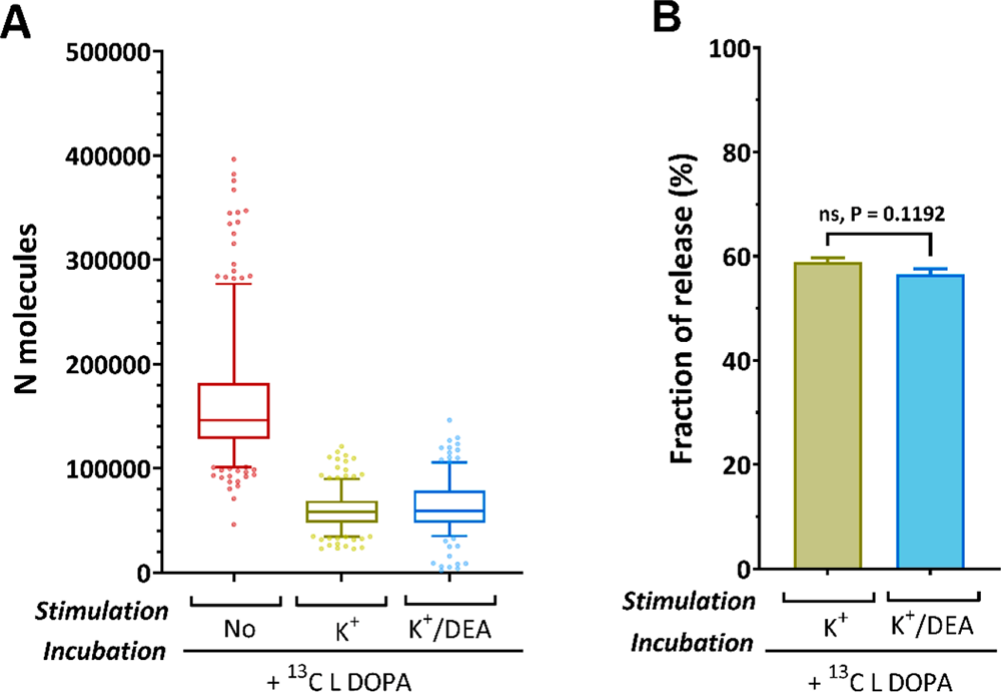
Comparison of the calculated *N*_molecules_ and the fraction of release. The *N*_molecules_ are calculated based on eq 2. *V*_vesicle_ is calculated using *d*_vesicle_ obtained from the vesicle size measurements with TEM (S2), assuming the vesicle is spherical. Finally, the fraction of release is calculated based on eq 7. Comparison of (A) the vesicular content from the non-stim group (^13^C L-DOPA, no K^+^ stim), stim K^+^ group (^13^C L-DOPA, K^+^ stim), and stim K^+^/DEA group (^13^C L-DOPA, K^+^/DEA stim). (B) Fraction of release calculated for the stim K^+^ and stim K^+^/DEA groups. For the stim K^+^/DEA group, only vesicles having ^127^I^–^/^12^C_2_^–^ ratios above the control level are used. The data set in A is presented with a box plot 25th–75th percentile, middle line 50th percentile, whisker boundary 5th–95th percentile. The data set in B is presented with column, mean ± standard error of the mean (SEM), and compared with a nonparametric, two-tailed Mann–Whitney unpaired test, * *P* < 0.05, ** *P* < 0.01, *** *P* < 0.001, **** *P* < 0.0001.

Figures 6A,B show the number of molecules *N*_molecules_ and the fraction of release (F%) calculated from the abundance measurement δ^13^C‰ and the vesicles size, respectively, applying the equations described above. The results show that an average vesicle releases approximately 60% of the total catecholamine during two stimuli in the labeled PC12 cells, which is close to the fraction previously reported by electrochemical techniques ranging between 40% and 65%.^24,30^ Additionally, we observed no significant difference in the fraction of release between cells stimulated with K^+^ alone and cells stimulated with K^+^/DEA (Figure 6B). To carefully assess if DEA has any effect on the release process, we also performed single-cell amperometry experiments (data is shown in S5). The results showed that ^127^I-DEA does not significantly change the amount of neurotransmitters released, but it reduces the number of exocytotic events during the second stimulus. This confirms that the use of ^127^I-DEA fits the scope of this work to use it as a second marker for partial release in NanoSIMS imaging and does not cause artifacts for single release events.

## Conclusions

In this work, we found direct evidence revealing partial release visually and quantitatively by implementing a nanoscopic approach with TEM and NanoSIMS mass spectrometry imaging. Colocalization of ^13^C and ^127^I^–^ in several vesicles was observed after incubation with ^13^C L-DOPA and ^127^I^–^ DEA in the stimulation solution. Thus, during partial release the ^127^I^–^ DEA was able to diffuse into the vesicles through the open fusion pore before the pore closed again, confirming the process of incomplete vesicle release through a transient fusion pore and providing a means to further evaluate it. The vesicles that underwent partial release were characterized by a size reduction, a decreased level of ^13^C-labeled dopamine together with an increased level of ^127^I. We were able to use a quantitative NanoSIMS approach to calculate the fraction of release directly from the abundance measurement δ^13^C‰ and vesicle size measurement, confirming data from electrochemical techniques, but here for individual vesicles instead of collections of vesicles. In addition to verifying and quantifying fractional release at single vesicles, this work unequivocally demonstrates that the process of partial release also provides an unexplored mechanism for molecular transport into cells via open fusion pores. This could have implications ranging from plasticity to medicine.

## Methods

### Single-Cell Preparation

PC12 cells (donated by Lloyd Greene lab, Columbia University) were cultured in RPMI-1640 medium (Lonza, Fisher Scientific, Sweden) supplemented with 10% donor equine serum and 5% fetal bovine serum in a 7% CO_2_, 100% humidity atmosphere at 37 °C. The culture medium was replaced with fresh medium every 2 days.

The stock solutions of l-3,4-dihydroxyphenylalanine (L-DOPA) were prepared by dissolving L-DOPA in purged Dulbecco’s phosphate-buffered saline without calcium and magnesium (Sigma-Aldrich, Sweden), in the dark and continuously purging with argon (6.0, AGA Sweden) to protect L-DOPA from oxidation. A final L-DOPA solution of 150 μM was obtained by diluting stock solution in warm cell media prior to incubation. Isotonic saline buffer contained 150 mM NaCl, 5 mM KCl, 1.2 mM MgCl_2_, 2 mM CaCl_2_, 5 mM glucose, 10 mM HEPES, pH 7.4 was prepared. The K^+^ stimulation solution contained 100 mM KCl, 55 mM NaCl, 1.2 mM MgCl_2_, 2 mM CaCl_2_, 5 mM glucose and 10 mM HEPES, pH 7.4. di-*N*-Desethyl amiodarone (DEA) (Santa Cruz Biotechnology, Dallas, Texas, USA) stock solution was prepared by dissolving 1 mg of DEA in 1 mL of 60% MeOH and kept in the dark. The final concentration of 28.3 μM DEA was obtained by diluting stock solution in stimulation solution or in an isotonic saline buffer bath around the cells.

For TEM - NanoSIMS imaging, PC12 cells were seeded on poly-d-lysine (Sigma) coated glass bottom dishes (50/30 mm diameter, Willco Wells B.V. The Netherlands) at a density of 10 000 cells per dish and cultured in 4–5 days prior to the experiments. Cells were then incubated for 6 h with 150 μM ^13^C L-DOPA (stable isotope-labeled l-3,4-dihydroxyphenylalanine (1–^13^C, RING-^13^C_6_, 99%, Cambridge Isotope Laboratories Inc., MA, USA)) in PC12 medium. In the case of stimulated cells, the cells were washed and kept in a warm isotonic saline buffer and exocytosis was induced by 100 mM KCl.

### Chemical Fixation and embedding

Cells plated on poly-d-lysine coated glass bottom dishes, right after incubation or incubation followed by stimulation, were washed with 0.1 M 1,4-piperazinediethanesulfonic acid (PIPES) buffer once and immediately primary fixed with prewarmed (37 °C) 2.5% glutaraldehyde in 0.1 M PIPES for 20 min at room temperature (RT). This was followed by washing 6 times with 0.1 M PIPES for 5 min each at RT. The fourth washing step included 50 mM glycine to block unreacted aldehydes. After the last wash, samples were stored at 4 °C until secondary fixation took place. Secondary fixation was performed with 1% osmium tetroxide in 0.1 M PIPES buffer for 30 min on ice in the dark. Before tertiary fixation with 1% uranyl acetate in water for 30 min on ice, samples were washed 6 times with water for 3 min each. Tertiary fixed samples were washed 3 times with water, after which dehydration with 30, 50, 70, 85, 95, and 100% ethanol (5 min each, three times 100% ethanol) was done on ice. Samples were infiltrated with 1:2 Agar100 resin (Agar Scientific):100% ethanol for 15 min at room temperature, after which the solution was changed to 2:1 Agar100:100% ethanol for another 15 min. Subsequently, solutions were exchanged three times with 100% Agar100 (5 min, 5 min, 10 min at RT). Finally, solutions were exchanged to Agar100 with accelerator BDMA (240 μL per 10 mL Agar100 mixed for at least 30 min on rotator) for 10 min. Dishes were inverted onto Agar100 with BDMA filled BEEM capsules, inverted again (capsule bottom-up) and polymerized at 60 °C for 16 h.

### Transmission Electron Microscopy

Monolayers of cells on the block surface were then cut into 300 nm thin sections with a Leica ultramicrotome EM UC6. Sections were placed on a copper finder grid with a carbon support film (Electron Microscopy Sciences, FCF200F1-CU). TEM imaging was performed using Talos Fei L120C (Thermo Scientific).

### NanoSIMS Imaging

NanoSIMS imaging was performed with a NanoSIMS 50L (CAMECA, France) at the Chemical Imaging Infrastructure at Chalmers University of Technology and University of Gothenburg on the same thin sections (300 nm) from which TEM images were acquired. These sections were then coated with a thin layer of Au (conductive material) to reduce sample charging during the analysis. Prior to each measurement, a saturation fluence of 10^17^ Cs^+^·cm^–2^ was implanted at the area of interest. A 16 keV Cs^+^ primary ions beam of ∼2 pA (D1_2) with the probe size of 150 nm was used to scan across the sample surface, providing secondary ion images of the ^12^C^14^N^–^, ^12^C_2_^–^, ^13^C^12^C^–^, and ^127^I^–^ ions. Images contained 8–10 cycles of 256 × 256 pixels, with a raster size of ∼20 μm × 20 μm, and a dwell time of 5 ms/pixel. Mass resolving power of 10 000 was obtained, which is sufficient to resolve potential mass interferences. ROIs were defined by manual thresholding based on vesicles features in the images.

### Data Processing

NanoSIMS images were processed using WinImage (CAMECA). Sequential image planes were collected, drift corrected, accumulated, and ratio images were generated (^13^C^12^C^–^/^12^C_2_^–^, ^127^I^–^/^12^C_2_^–^). Ratio images of ^13^C^12^C^–^/^12^C_2_^–^ were presented in δ‰ scale. Overlay images of TEM and NanoSIMS were produced using ImageJ. For further evaluation of the isotopic enrichment in samples, count rates and ratios of the selected ion species were extracted from designated ROIs on drift corrected and dead-time corrected layer images.

The results for isotopic ratios are expressed as deviations from a reference isotopic ratio VPDB in ‰ (per mille) were generated using the following equation:
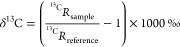
where the reference value ^^13^C^*R*_VPDB_ = ^13^C/^12^C = 0.0112372 (VPDB, Vienna Pee Dee Belemnite)
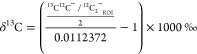


## Supporting Information Available

S1. Supplemental images. Figure S1.1. NanoSIMS ion images of ^12^C_2_^–^, ^13^C^12^C^–^, ^12^C^14^N^–^, ^127^I^–^. Figure S1.2. TEM and NanoSIMS ratio images of three represent single cells that were incubated with ^13^C L-DOPA for 6 h and stimulated with K^+^/DEA. Figure S2. Vesicle size analysis. S2. Vesicle size. S3. Vesicle spatial segregation. Figure S3.1. Representative TEM images from 300 nm-thick sections from the control group, non-stim group, stim K+ group, and stim K+/DEA group. Figure S3.2. Localization of ^13^C-dopamine enriched vesicles in different samples. S.4. Validation of partial release with NanoSIMS data from freeze-dried samples. Figure S4.^13^C enrichment drop upon K^+^ stimulation observed in freeze-dried samples. S.5. Assessing the effects of di-*N*-desethyl amiodarone on the release process using single-cell amperometry. Figure S5.1. Single-cell amperometry reveals ^127^I-DEA does not affect the amount of exocytotic release while it reduces the number of exocytotic events. Figure S5.2. Single-cell amperometry peak analysis for the first and the second stimulation with K^+^ and K^+^/DEA. Parameters include half peak width (*t*_1/2_), peak current (*I*_max_), rise time (t_rise_), fall time (*t*_fall_), prespike foot current (*I*_foot_), and foot time (*t*_foot_) (PDF)
