# Supplementary material for: Visualization of Partial Exocytotic Content Release and Chemical Transport into Nanovesicles in Cells
Source: ACS Nano. 2022 Feb 21;16(3):4831–42. doi: 10.1021/acsnano.2c00344 (PMC8945366; doi:10.1021/acsnano.2c00344)
Supplement: Supplementary file 1 — nn2c00344_si_001.pdf [file nn2c00344_si_001.pdf]

Supporting information

Visualization of partial exocytotic content release and chemical transport into nanovesicles in cells

Tho Duc Khanh Nguyen[1], Lisa Mellander[1], Alicia Lork[1], Aurélien Thomen[1], Mai Philipsen[2], Michael E. Kurczy[3], Nhu T.N. Phan[1], Andrew G. Ewing[1]*

[1]Department of Chemistry and Molecular Biology, University of Gothenburg, Gothenburg SE-412 96, Sweden;
[2]Department of Chemistry and Chemical Engineering, Chalmers University of Technology, Gothenburg SE-412 96, Sweden;
[3]Cardiovascular and Metabolic Diseases, Innovative Medicines and Early Development Biotech Unit, AstraZeneca, Mölndal SE-431 83, Sweden

Contents

S.1. Supplemental images 2

S.2. Vesicles size analysis 3

S.3. Vesicle spatial segregation 4

S.4. Validation of partial release with NanoSIMS data from freeze-dried samples 5

S.5. Assessing the effects of di-N-desethyl amiodarone on the release process using single-cell amperometry 6

References 10

S.1. Supplemental images

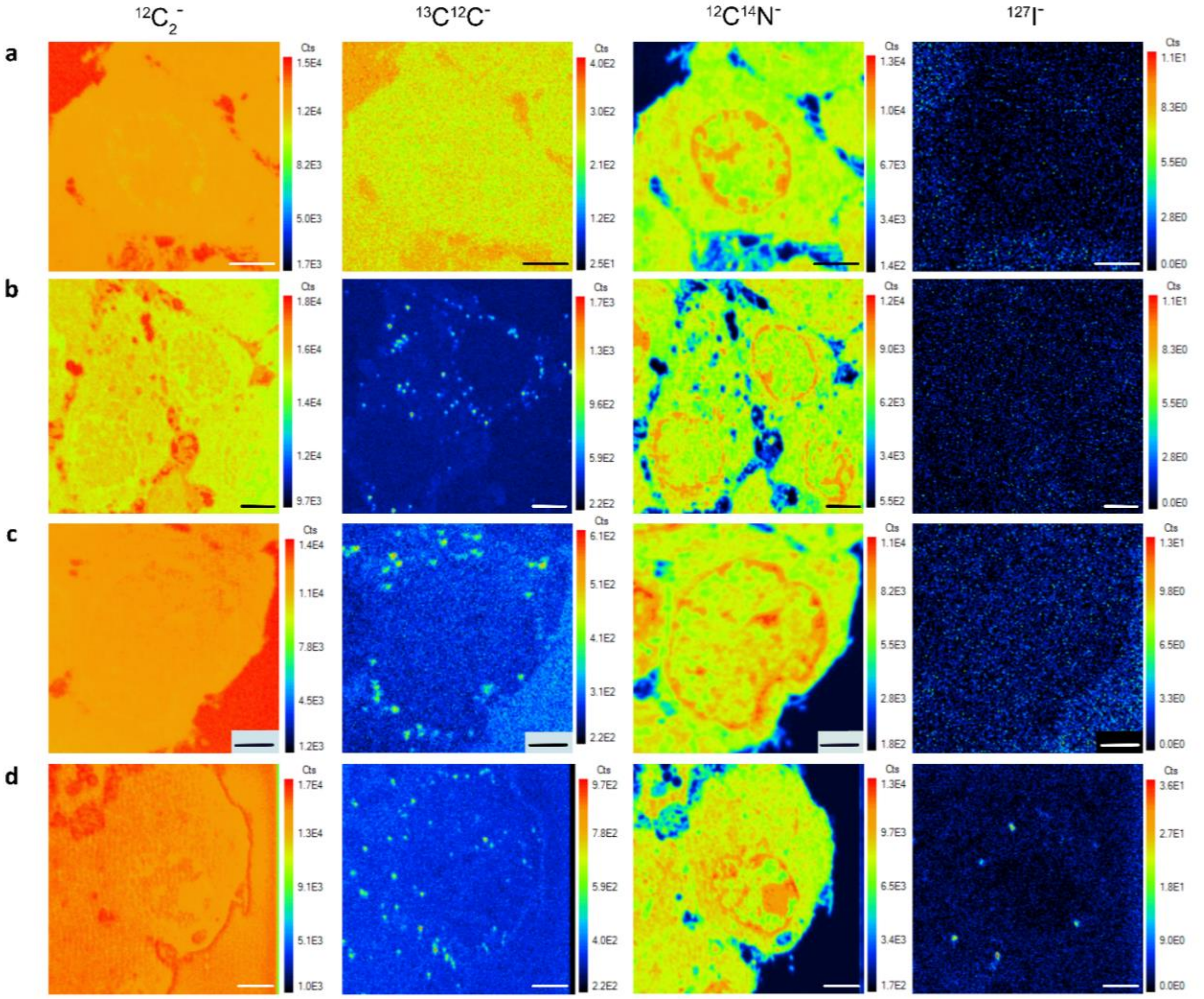

Figure S1.1. NanoSIMS ion images of $^{12}C_2^-$, $^{13}C^{12}C^-$, $^{12}C^{14}N^-$, $^{127}I^-$ used to generate ratio images in fig 2. The panels a-d correspond to the same in Figure 2.

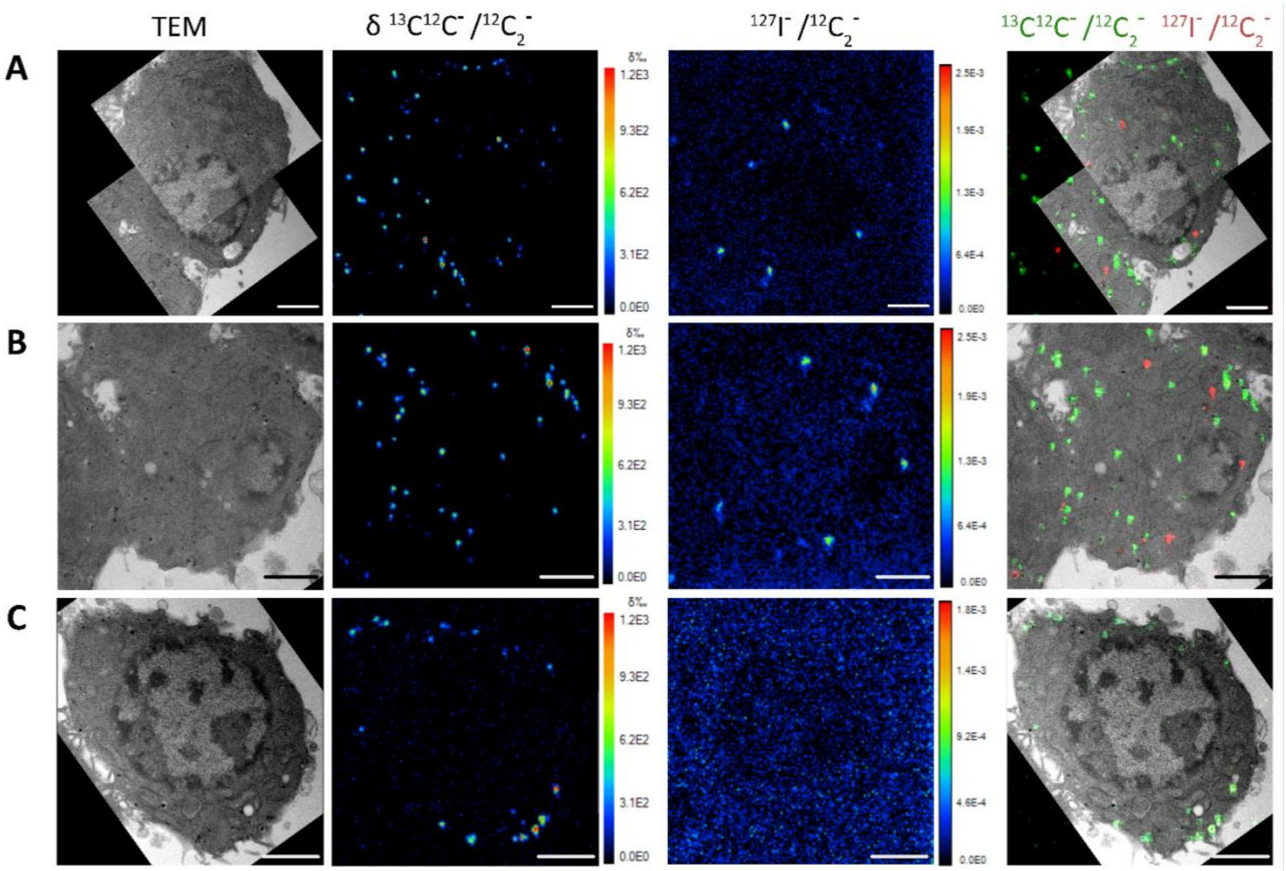

Figure S1.2. TEM and NanoSIMS ratio images of three representative single cells that were incubated with ^{13}C L-DOPA for 6 h and stimulated with K^+/DEA . A-C are three different cells.

S.2. Vesicles size analysis

Size analysis was performed on TEM images of the 300-nm sections used to acquire NanoSIMS images, with ImageJ (https://imagej.nih.gov/ij/) by the tool Analyze\Measure. The long axes of the structures (dense-core and vesicle membrane) were measured. Data in Table S1 were presented in mean ± SEM. $n_{sections}$= 4 for each group (cells with clear TEM images define dense-core and vesicle membrane were used). Data for inner vesicles were collected from $N_{vesicles}$= 54, 124, 88, 45 for no L-DOPA, no stim; L-DOPA, no stim; L-DOPA stim K^+; L-DOPA stim K^+/DEA, respectively. Data for outer vesicles were collected from $N_{vesicles}$= 69, 144, 112, 120 for no L-DOPA, no stim; L-DOPA, no stim; L-DOPA stim K^+; L-DOPA stim K^+/DEA, respectively.

Table S2. Vesicles sizes from TEM images

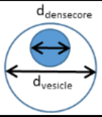		Inner		Outer	
		Densecore (nm)	Vesicle (nm)	Densecore (nm)	Vesicle (nm)
No ^{13}C L-DOPA	No stim	92 ± 7	138 ± 11	107 ± 4	153 ± 7
Loaded ^{13}C L-DOPA	No stim	100 ± 5	225 ± 12	100 ± 6	222 ± 12
	Stim K^+	93 ± 6	188 ± 14	93 ± 6	155 ± 10
	Stim K^+, DEA	109 ± 12	221 ± 22	113 ± 5	176 ± 8

S.3. Vesicle spatial segregation

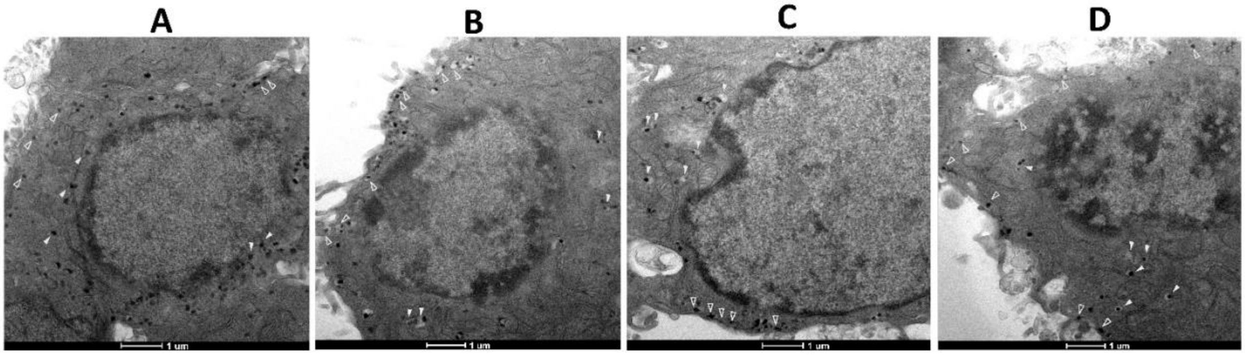

Figure S3.1. Representative TEM images from 300-nm-thick sections of (A) control group (no ^{13}C L-DOPA incubation, no K^+ stimulation), (B) non-stim group (^{13}C L-DOPA incubation, no K^+ stimulation), (C) stim K^+ group (^{13}C L-DOPA incubation, K^+ stimulation) and (D) stim K^+/DEA group (^{13}C L-DOPA incubation, K^+ stimulation in the presence of DEA) showing that vesicles distribute more inside or equally across the non-stimulated cells, whereas they tend to reside near the cellular plasma membrane in the stimulated cells.

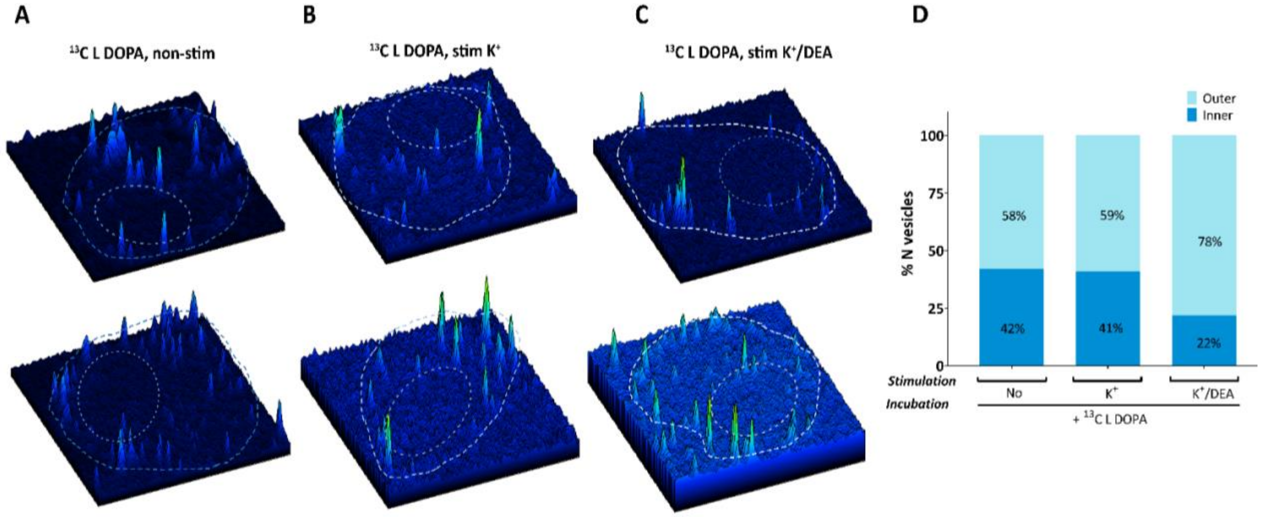

Figure S3.2. Localization of ^{13}C dopamine enriched vesicles in different samples. Representative surface plots from NanoSIMS ratio images of $^{13}C^{12}C^-/^{12}C_2^-$ for PC12 cells incubated with ^{13}C L-DOPA for 6 h. (A) no stimulation, (B) stimulation with K^+, (C) stimulation with K^+/DEA. The dash lines mark the nucleus and plasma membrane. (D) The average percentage number of vesicles located at the outer (within a two-vesicle diameter to the plasma membrane) versus the inner part of the cells. DEA shown to facilitate vesicle transport toward the plasma membrane. Surface plots were produced using the ImageJ (https://imagej.nih.gov/ij/) where the $^{13}C^{12}C^-/^{12}C_2^-$ were plotted on the z-axis (Color scales were different in different images; therefore, they do not represent the same enrichment range).

S.4. Validation of partial release with NanoSIMS data from freeze-dried samples

For freeze-drying – NanoSIMS, PC12 cells were seeded on Poly-D-Lysine (Sigma) coated Si wafer (5×5mm, Si-Mat, Silicon Materials, Kaufering, Germany) and cultured 4-5 days prior to the experiments. Cells were then incubated 1.5 h or 3 h with 150 μM ^{12}C L-DOPA in PC12 cell medium. The cells were washed twice and kept in a warm isotonic saline buffer. In the case of stimulated cells, exocytosis was induced by 100 mM KCl.

The cells were plated on Si wafers and after incubation/incubation - stimulation were fixed with 2.5 % Glutaldehyde in DPBS for 15 min at room temperature (RT), then rinsed with Mili Q water twice to remove salts. The cells were then snap-frozen at -160 °C by plunging into liquid nitrogen–chilled isopentane and dried under a vacuum chamber overnight. The samples were maintained in a vacuum desiccator until NanoSIMS analysis.

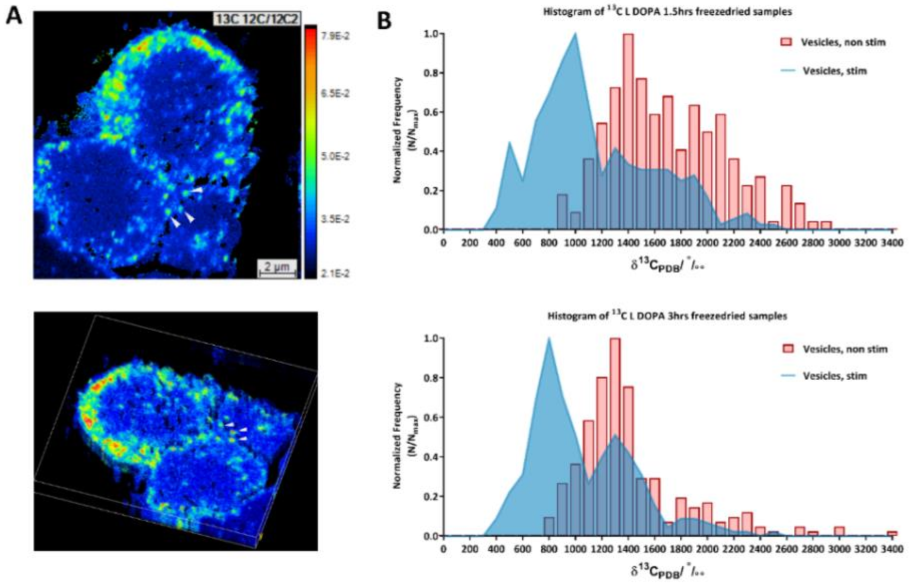

Figure S4. ^{13}C enrichment drop upon K^+ stimulation observed in freeze-dried samples: **(A)** Representative NanoSIMS ratio images of $^{13}C^{12}C^-/^{12}C_2^-$ (accumulated 50 cycles) for PC12 cells, incubated with ^{13}C L-DOPA for 1.5 h reveals vesicles enriched with ^{13}C dopamine (example vesicles marked with white triangles), top panel: 2D view, bottom panel: 3D view; **(B)** Normalized histograms of all ROIs (vesicles) from freeze-dried PC12 cells labeled via incubation with ^{13}C L-DOPA for 1.5 h (top) and 3 h (bottom), with and without K^+ stimulation, bin size: 100 $\delta^{13}C_{PDB}$ (‰), n=7 cells for each condition (total 7×4 cells).

The histograms reveal the drop in ^{13}C dopamine enrichment in the stimulated cells compared to non-stimulated cells, which is in good agreement with the NanoSIMS data acquired from resin embedded samples. It should be noted that the enrichment range of ^{13}C dopamine generally is higher in the freeze-dried samples compared to that in resin embedded samples due to the concentration effect. This in the freeze-dried sample owing to compaction of the cell after the procedure, whereas in the resin embedded sample, the aqueous environment of the cell is replaced by the resin, maintaining the shape of the cell without shrinking.

S.5. Assessing the effects of di-N-desethyl amiodarone on the release process using single-cell amperometry

Cell culture

PC12 cells were seeded on collagen-coated culture dishes (60 mm diameter, collagen type IV, BD Biosciences, Bedford, MA) at a density of 10000 cells per dish and cultured in 4-5 days prior to the experiments. Cells were then incubated for 1.5 h with 150 µM ^{12}C L-dopa (Sigma-Aldrich, Sweden) in PC12 medium. The cells were washed twice to remove the culture medium and kept in a warm isotonic saline buffer (37°C) during the SCA experiment. Exocytosis was induced by 100 mM KCl.

Fabrication of disk-shaped carbon-fiber microelectrodes

Carbon fiber disk microelectrodes were fabricated as previously described.[1] Electrodes were tested in a solution of 100 µM dopamine in PBS (pH 7.4) with cyclic voltammetry (-0.2 to 0.8 V vs Ag/AgCl, 100 mV/s). Microelectrodes with steady-state voltammetric behavior were used for single-cell amperometry measurements.

Single-cell amperometry

Single-cell amperometry measurements were performed on an inverted microscope (IX81, Olympus) in a Faraday cage. An Ag/AgCl reference electrode was placed in the isotonic saline bath surrounding the cells. The working electrode was held at +700 mV versus the reference electrode with an Axopatch 200B potentiostat (Molecular Devices; Sunnyvale, CA). The output from the potentiostat was digitized at 5 kHz and filtered at 2 kHz. For electrochemical recording, a 5 µm carbon fiber disk electrode was carefully placed onto a single PC12 cell. A glass micropipette containing K^+ stimulation solution with or without DEA was then positioned close to the cell. Each cell was stimulated twice with a 5 s injection pulse (20 psi) via a micropipette connected to a microinjection system (Picospritzer II; General Valve Corporation, Fairfield, NJ). The time interval between each stimulus was 2 min to make sure the cell calcium level was reduced to the basal level prior to the second stimulation.[2]

Dara processing

The electrochemical data were processed with Igor Pro 6.37 (David Sulzer's group, Columbia University). The filter that was used for the current was 1 kHz (binomial sm.). The threshold to detect main peaks and pre-spike foot were set at five times and two times the standard deviation of the noise, respectively. After peak detection, all peaks in amperometric traces were inspected and false positives were manually rejected. For further analysis, the mean of medians $N_{molecules}$, N_{events} , I_{max}, t_{half}, t_{rise} and t_{fall} from each single cell was used to minimize the impact of extreme values. Datasets were compared with a nonparametric, two-tailed Mann Whitney unpaired test, * P < 0.05, ** P < 0.01, *** P < 0.001, **** P< 0.0001.

SCA experiments were carried out to investigate whether ^{127}I-DEA caused any effect on the amount of exocytotic release. The released neurotransmitters were oxidized immediately when they were released and diffused to the electrode surface, generating a current response in function of time, a so-called spike. Therefore, analyzing the shape of the spikes (e.g., t_{rise}, t_{half}, t_{fall}, *etc.*) provides essential

information about the release event dynamics. In addition, the number of molecules released (N) can be quantified based on the charge (Q) (Figure S5.1.A).

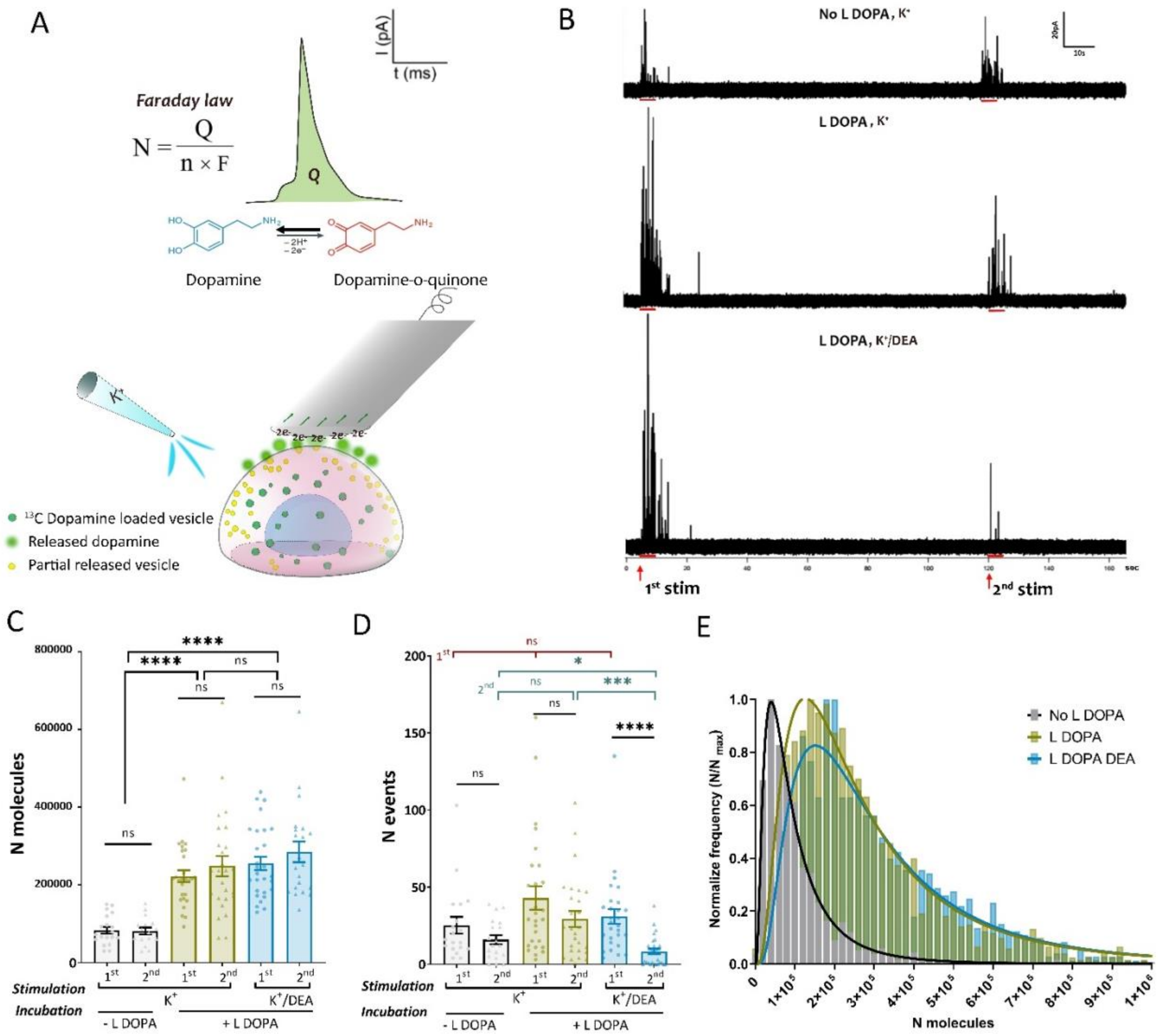

Figure S5.1. Single-cell amperometry reveals ^{127}I-DEA does not affect the amount of exocytotic release while it reduces the number of exocytotic events in a second round of stimulations. **(A)** Schematics illustrates single-cell amperometry; **(B)** Representative SCA amperometric traces of exocytosis from single PC12 cells stimulated with 2 consecutive 100 mM K^+ stimuli. Traces represent the release from single cells belonging to the control group (without L-DOPA loaded), L-DOPA loaded group and L-DOPA loaded and stimulated in the presence of DEA, top to bottom, respectively. The red line underneath each trace indicates the stimulus time. **(C)** The amount of exocytotic release ($N_{molecules}$) and **(D)** the average number of exocytotic events per cell (N_{events}) for the first and second stimulation. Data represent means ± SEM. Datasets were compared with a nonparametric, two-tailed Mann Whitney unpaired test, * $P < 0.05$, ** $P < 0.01$, *** $P < 0.001$, **** $P < 0.0001$. **(E)** Normalized histograms of all spikes, bin size: 20000 molecules, fit curves: log-normal distributions. The number of cells used for the control group (No L-DOPA) was 19, for L-DOPA loaded group was 27, and for L-DOPA loaded, stimulated in the presence of DEA was 28. The numbers of exocytotic events analyzed were 782, 1950, and 1087, respectively, for the 3 groups. The experiments were carried out on 3 consecutive generations of PC12 cells.

As a previous study showed no significant difference in the number of molecules released between PC12 cells treated with ^{12}C- or ^{13}C L-DOPA,[3] we performed all electrochemical experiments by treating the cells with ^{12}C L-DOPA. Typical amperometric traces obtained are shown in Figure S5.1.B. Each trace represents a series of spikes following each stimulus, in which each spike corresponds to a single vesicle release event.

Evaluating the amount of exocytotic release ($N_{molecules}$), as expected, Figure S5.1.C shows a significant increase between cells treated with L-DOPA (for both groups stim by K^+ and stim by K^+/DEA) compared to the control cells without L-DOPA treatment. In addition, between the two groups that were both treated with L-DOPA, cells stimulated with K^+/DEA released slightly more than those stimulated with only K^+; however, the difference is not statistically significant. When comparing the number of molecules released between the first and the second stimulation, there were no significant differences for all the groups of cells. Detailed SCA peak analysis data is shown in Figure S5.2.

The numbers of release events are shown in Figure S51.D. The results show a reduction in the number of spikes indicating that the number of exocytotic release events per cell decreases upon repetitive stimuli (Figure S5.1.D), consistent with a previous study on repetitive stimulation.[2] In addition, a significant decrease in the number of events appeared in the second stimulation for the stim K^+/DEA group. We speculated this occurs from ^{127}I-DEA anchoring deeply into the lipid bilayer,[4–6] reducing phospholipid mobility needed for vesicles to rearrange their membrane lipid to form a fusion pore needed for a release event.

These comparisons show that ^{127}I-DEA does not significantly change the amount of neurotransmitters released, but only it reduces the number of exocytotic events during the second stimulus. This confirms that the use of ^{127}I-DEA fits the scope of this work to use it as a second marker for partial release in NanoSIMS imaging and does not cause artifacts for single release events.

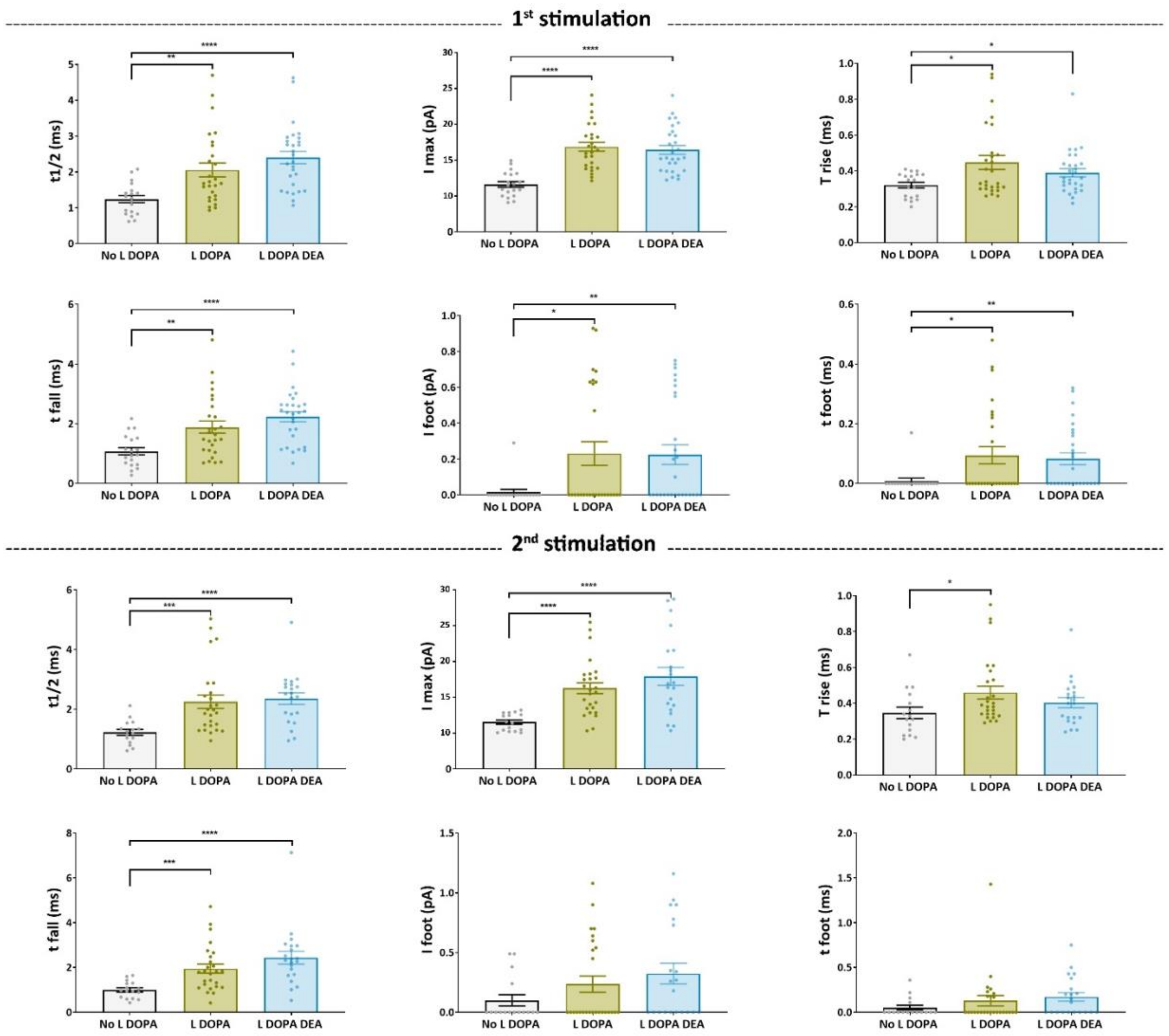

Figure S5.2. Single-cell amperometry peak analysis for the first and the second stimulation with K^+ and K^+/DEA. Parameters including half peak width ($t_{1/2}$), peak current (I_{max}), rise time (t_{rise}), fall time (t_{fall}), pre-spike foot current (I_{foot}), foot time (t_{foot}). Data represent means ± SEM. Datasets were compared with a nonparametric, two-tailed Mann Whitney unpaired test, * $P < 0.05$, ** $P < 0.01$, *** $P < 0.001$, **** $P< 0.0001$. The number of cells used for control group (No L-DOPA) was 19, for L-DOPA loaded group was 27, and for L-DOPA loaded, stimulated in the presence of DEA was 28. The numbers of exocytotic events analyzed were 782, 1950 and 1087, respectively, for the 3 groups. The experiments were carried out on 3 consecutive generations of PC12 cells.

References

(1) Adams, K. L.; Engelbrektsson, J.; Voinova, M.; Zhang, B.; Eves, D. J.; Karlsson, R.; Heien, M. L.; Cans, A.-S.; Ewing, A. G. Steady-State Electrochemical Determination of Lipidic Nanotube Diameter Utilizing an Artificial Cell Model. *Anal. Chem.* **2010**, *82*, 1020—1026.

(2) Gu, C.; Larsson, A.; Ewing, A. G. Plasticity in Exocytosis Revealed through the Effects of Repetitive Stimuli Affect the Content of Nanometer Vesicles and the Fraction of Transmitter Released. *Proc. Natl. Acad. Sci.* **2019**, *116*, 21409 LP – 21415.

(3) Lovrić, J.; Dunevall, J.; Larsson, A.; Ren, L.; Andersson, S.; Meibom, A.; Malmberg, P.; Kurczy, M. E.; Ewing, A. G. Nano Secondary Ion Mass Spectrometry Imaging of Dopamine Distribution Across Nanometer Vesicles. *ACS Nano* **2017**, *11*, 3446–3455.

(4) Chatelain, P.; Ferreira, J.; Laruel, R.; Ruysschaert, J. M. Amiodarone Induced Modifications of the Phospholipid Physical State: A Fluorescence Polarization Study. *Biochem. Pharmacol.* **1986**, *35*, 3007–3013.

(5) Jiang, H.; Passarelli, M. K.; Munro, P. M. G.; Kilburn, M. R.; West, A.; Dollery, C. T.; Gilmore, I. S.; Rakowska, P. D. High-Resolution Sub-Cellular Imaging by Correlative NanoSIMS and Electron Microscopy of Amiodarone Internalisation by Lung Macrophages as Evidence for Drug-Induced Phospholipidosis. *Chem. Commun.* **2017**, *53*, 1506–1509.

(6) Mehraein, F. A Review on Amiodarone as an Antiarrhythmic Drug. *Abnorm. Hear. Rhythm.* **2015**, 115.
